# Integrating natural language processing into radiation oncology: a practical guide to transformer architecture and large language models

**DOI:** 10.1093/bjrai/ubaf010

**Published:** 2025-08-13

**Authors:** Reza Khanmohammadi, Mohammad M Ghassemi, Kyle Verdecchia, Ahmed I Ghanem, Bing Luo, Indrin J Chetty, Hassan Bagher-Ebadian, Farzan Siddiqui, Mohamed Elshaikh, Benjamin Movsas, Kundan Thind

**Affiliations:** Department of Computer Science and Engineering, Michigan State University, East Lansing, MI 48824, United States; Department of Computer Science and Engineering, Michigan State University, East Lansing, MI 48824, United States; Department of Radiation Oncology, Henry Ford Health, Detroit, MI 48202, United States; Department of Radiation Oncology, Henry Ford Health, Detroit, MI 48202, United States; Alexandria Department of Clinical Oncology, Alexandria University, Alexandria 21561, Egypt; Department of Radiation Oncology, Henry Ford Health, Detroit, MI 48202, United States; Department of Radiation Oncology, Cedars Sinai Medical Center, Los Angeles, CA 90048, United States; Department of Radiation Oncology, Henry Ford Health, Detroit, MI 48202, United States; Departments of Radiology and Osteopathic Medicine, Michigan State University, East Lansing, MI 48824, United States; Department of Physics, Oakland University, Rochester, MI 48326, United States; Department of Radiation Oncology, Henry Ford Health, Detroit, MI 48202, United States; Department of Radiation Oncology, Henry Ford Health, Detroit, MI 48202, United States; Department of Radiation Oncology, Henry Ford Health, Detroit, MI 48202, United States; Department of Radiation Oncology, Henry Ford Health, Detroit, MI 48202, United States; Department of Medicine, Michigan State University, East Lansing, MI 48824, United States

**Keywords:** artificial intelligence, radiation oncology, natural language processing, large language models, personalized medicine

## Abstract

Natural language processing (NLP) is a key technique for developing medical artificial intelligence (AI) systems that leverage electronic health record data to build diagnostic and prognostic models. NLP enables the conversion of unstructured clinical text into structured data that can be fed into AI algorithms. The emergence of transformer architecture and large language models (LLMs) has led to advances in NLP for various healthcare tasks, such as entity recognition, relation extraction, sentence similarity, text summarization, and question-answering. In this article, we review the major technical innovations that underpin modern NLP models and present state-of-the-art NLP applications that employ LLMs in radiation oncology research. However, it is crucial to recognize that LLMs are prone to hallucinations, biases, and ethical violations, which necessitate rigorous evaluation and validation prior to clinical deployment. As such, we propose a comprehensive framework for assessing the NLP models based on their purpose and clinical fit, technical performance, bias and trust, legal and ethical implications, and quality assurance prior to implementation in clinical radiation oncology. Our article aims to provide guidance and insights for researchers and clinicians who are interested in developing and using NLP models in clinical radiation oncology.

## Introduction

Artificial intelligence (AI) is transforming healthcare by improving patient outcomes, optimizing clinical workflows, and reducing costs.^1^ A key area where AI is rapidly evolving is precision medicine, which tailors personalized medical care to patients’ genes, environments, and lifestyles.^2^ Research in machine learning-based algorithms for the detection of disease-causing genetic mutations^3^ and individualized treatment protocols for patients^4^ is being developed and tested. Advanced AI models are being constructed within medicine for personalized disease prevention, care, and even prophylaxis. The ongoing trend of increased healthcare data collection will further aid innovative applications of these technologies in the future.

Natural language processing (NLP) is a subclass of AI that enables machines to understand and interpret human language. The goal of NLP is to develop algorithms and models that are capable of processing, analysing, and generating natural language text and speech. The history of natural language processing dates back to the 1950s when early language translation programs were developed.^5^ However, limited computing power and lack of data slowed progress.^6^ The introduction of statistical methods in the 1980s led to more sophisticated language models.^7^ The development of neural networks and deep learning in the 2010s led to significant advancements in NLP, culminating in the development of the famous transformer architecture in 2017.^8^ With the capabilities of the transformer architecture, a new breed of models emerged, referred to as large language models (LLMs). Notable LLMs include Bidirectional Encoder Representations from Transformers (BERT),^9^ GPT-4, and ChatGPT. The development of these technologies has accelerated language processing and opened new possibilities for interacting with machines using natural language. Despite these advancements, LLMs are not without limitations, including biases that may lead to inequitable predictions and hallucinations where models produce incorrect or nonsensical outputs. These issues highlight the importance of an effective evaluation framework to ensure the reliability and fairness of these models in clinical applications.

The transformer architecture has paved the way for the development of LLMs, which are now at the forefront of NLP research. Language modelling,^10^ machine translation,^11^ and sentiment analysis^12^ have been shown to perform well with these models. However, training such models is a complex and resource-intensive process requiring large size of data and computing. Researchers have explored the use of transfer learning to address this challenge,^13^ a technique that allows pre-trained models to be fine-tuned on specific tasks with limited data. Indeed, the real-world applications of NLP often begin with fine-tuning these pre-trained models to suit the specific needs of the task. Furthermore, zero-shot learning has also emerged as a promising method for generalizing new tasks without explicit training.^14^ This results in easy deployment, albeit evaluating model performance is becoming more challenging as input data has turned out to be more expansive and complex.^15^ As a result, researchers and practitioners in the field are facing a significant challenge as they must find ways to accurately assess the quality of these models when applied to prospective real-world data.^16^

Adequate testing of these models will be an issue in radiation oncology, which utilizes cutting-edge technology to guide precise radiation to the target cancer. Language processing and research applications in radiation oncology have been discussed in the literature.^17–22^ Highlights include enhanced insights into disease staging, treatment options, and patient outcomes,^23^ entity extraction from unstructured clinical notes^24^^,^^25^ to aid clinical decision support. The lack of clinical evaluation and proper validation for many of these models poses a significant challenge to their widespread adoption. The clinical evaluation of these models involves rigorous testing based on well-defined metrics and benchmark datasets. This includes assessing the potential risks and benefits in the prospective use of these models and a thorough evaluation of the impact on patient outcomes. The application of NLP models to the field of clinical radiation oncology has the potential to enhance its outcomes and efficiency, contingent upon the models’ ability to exhibit their validity, safety, fairness, and reliability as quantified by a rigorous evaluation framework. This requires collaboration between clinicians, data scientists, and regulatory bodies to develop detailed and robust evaluation frameworks that can ensure that these models are integrated into clinical workflows safely and effectively.^26^

In the sections below, we review the transformer architecture that has been foundational to the development of LLMs. We also provide an overview of training and fine-tuning for LLMs and present recent research applications in radiation oncology. Lastly, we will identify the existing challenges and limitations for the clinical adoption of LLMs and propose a framework that serves as a preliminary guideline to facilitate the safe, fair, and effective application of these algorithms in clinical settings. A glossary of technical terms is provided in Table 1 to assist readers in understanding the computational aspects discussed in the following section.

**Table 1.** Glossary of technical terms.

Term	Explanation
Natural Language Processing (NLP)	A field of artificial intelligence focused on enabling machines to understand, interpret, and generate human language.
Large Language Models (LLMs)	Advanced neural network models designed to process and generate text by analysing sequences of words, often with billions of parameters.
Transformer Architecture	A neural network architecture that uses self-attention mechanisms to process sequential data efficiently, allowing for parallel computation and long-range dependency modelling.
Parameters (for LLMs)	Numerical values in a neural network that are learned during training and define the behaviour of the model. LLMs can have billions (eg, 1B-405B) of parameters.
Tokenization	The process of breaking down text into smaller units, such as words or subwords, for model input.
Embedding	The representation of words or tokens as dense numerical vectors, capturing semantic meaning.
Positional Encoding	Adds information about the order of tokens in a sequence, allowing transformers to process sequential data effectively.
Transfer Learning	A method where pre-trained models are adapted for specific tasks by fine-tuning on task-specific data.
Fine-Tuning	Adjusting the parameters of a pre-trained model on a smaller, domain-specific dataset to improve performance for specific tasks.
Zero-Shot Learning	The ability of a model to perform tasks without explicit training on them, relying on pre-trained knowledge.
Few-Shot Learning	The ability of a model to learn and generalize from a small amount of labelled data for a new task.
Encoder-Only Models	Models like BERT that analyse entire input texts at once and excel in context-based tasks.
Decoder-Only Models	Models like GPT that generate text by predicting the next word based on preceding words.
Encoder-Decoder Models	Models like T5 and BART that combine encoding and decoding capabilities for both understanding and generating text.
Named Entity Recognition (NER)	An NLP task where specific entities (eg, names, dates) are identified and classified in text.
Self-Attention Mechanism	A technique used in transformers that calculates the relevance of each word in a sequence to every other word, enabling context understanding.
Scaled Dot-Product Attention	A mathematical operation in transformers that calculates the importance of tokens relative to others in a sequence.
Residual Connections	Techniques used in neural networks to improve gradient flow and training stability by adding the input of a layer to its output.
Layer Normalization	A method to stabilize and accelerate training by normalizing layer inputs.
Adversarial Testing	A technique to evaluate model robustness by testing on edge cases or perturbed inputs.
BLEU (Bilingual Evaluation Understudy)	A metric used to evaluate the quality of machine-generated text by comparing it to human-generated references.
Rouge Metrics	Metrics that evaluate the overlap between machine-generated and human-generated summaries to assess summarization quality.
Hugging Face Transformers Library	A popular open-source library for NLP that provides pre-trained models and tools for fine-tuning.
Google TensorFlow	An open-source machine learning framework for building and deploying AI models.
Meta PyTorch	An open-source machine learning library known for its flexibility and efficiency in building deep learning models.

## Materials and methods

### Literature review

We employed a 2-pronged approach to the literature review to ensure a comprehensive review of modern NLP techniques and their applicability in radiation oncology. First, we utilized ArXiv to capture the most recent advancements in the NLP field, recognizing that cutting-edge research is often disseminated through this preprint server before formal peer review. Search terms used on ArXiv included combinations of “Natural Language Processing,” “Transformer Architecture,” “Large Language Models,” and “Clinical Applications.” Subsequently, we explored PubMed to obtain NLP research applications in Radiation Oncology. Our search terms for PubMed encompassed “Natural Language Processing,” “Radiation Oncology,” “Electronic Health Records,” and “Clinical AI Applications.” This literature was critical for summarizing the foundational elements of modern NLP pipelines and their use in radiation oncology research, as well as for considerations for the design of the implementation framework of these models in clinical radiation oncology. Below, we summarize the key foundational elements of the modern NLP models.

### Recurrent neural networks

Recurrent neural networks (RNNs) were foundational in early NLP for processing sequential data and capturing temporal dynamics. However, they struggle with long-term dependencies due to vanishing or exploding gradients.^27^ Long short-term memory (LSTM) networks were introduced to address these limitations with mechanisms like gating and internal feedback loops, enhancing their ability to retain and learn long-term dependencies. LSTMs became the backbone for various NLP tasks such as machine translation and sentiment analysis. Despite these improvements, LSTMs faced challenges with sequential computation, limiting parallelization and efficiency. This led to the development of the transformer architecture, which enables parallel computation and utilizes self-attention mechanisms to capture long-range dependencies more effectively.^8^

### Transformer architecture

The transformer architecture, introduced in the 2017 paper “Attention is All You Need” by Vaswani et al,^8^ revolutionized NLP by replacing RNNs with self-attention mechanisms, enabling parallel computation and capturing long-range dependencies more effectively. The transformer comprises an encoder and a decoder: the encoder processes the input sequence into a context-rich representation, which the decoder uses to generate the output.^28^

Transformers surpass previous NLP models like LSTM in 2 major ways: they excel at contextual learning via multiheaded attention,^29^ and they facilitate parallelized computations, speeding up training.^30^ The transformer processes input text by breaking it into tokens, which are converted into dense vector representations through input embedding. Positional encoding is added to these embeddings to maintain the order of the tokens.

**Figure 1.**
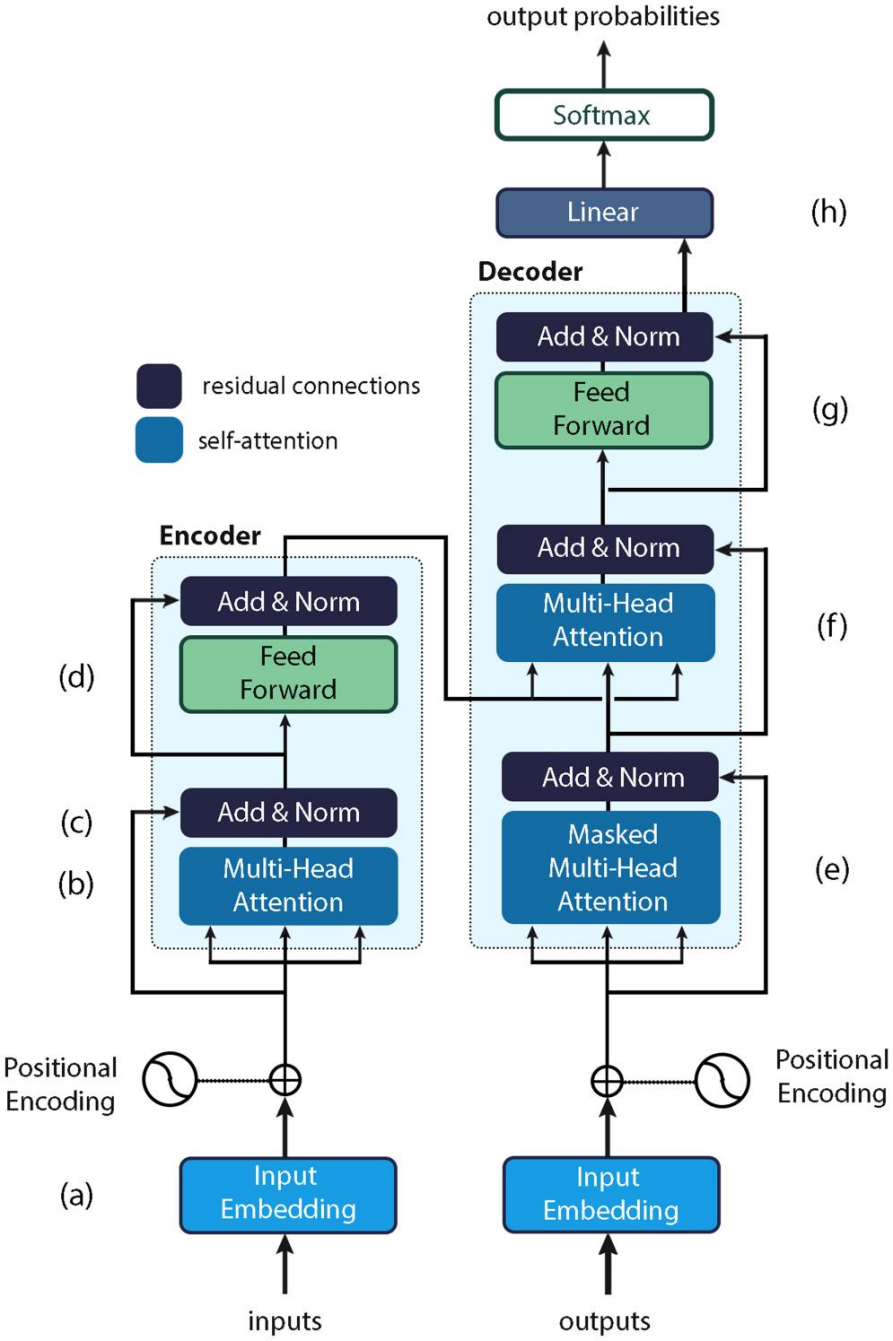
Vaswani et al^8^ illustration of the transformer architecture. First, input words are embedded and encoded based on their position in the input sequence (a). In the encoder, the Multi-head Attention enables each word to attend to other words in the sequence, capturing relationships and dependencies (b). Then, the original input embeddings are added to the self-attention outputs and normalized to integrate attended information while preserving the input (c). Next, a neural network captures complex patterns and interactions (d) before representations are added and normalized. On the other hand, the decoder’s masked self-attention allows the decoder to capture dependencies among its own outputs (e). The decoder’s second self-attention (f) attends to the encoded input sequence, allowing it to access the information from the input and align it with the current decoding position. Similar to the encoder, outputs are further processed with another feed-forward neural network (g), added, and normalized. Finally, the decoder applies a linear transformation (h) followed by a softmax activation function to generate the probability distribution over the vocabulary to select the next token.

As shown in Figure 1, the architecture consists of layers with 2 main components: a self-attention mechanism and a feed-forward network. The self-attention mechanism calculates attention scores between tokens, normalizes them with a softmax function, and computes a weighted sum of the input sequence, emphasizing important segments. The feed-forward network refines the output, with each component augmented by residual connections and layer normalization to ensure robust gradient flow during training.

Using a scaled dot-product attention mechanism, the transformer captures long-range dependencies by calculating similarity scores between tokens, normalizing them, and weighting the input sequence accordingly. This process, repeated across multiple layers, allows the transformer to derive context and has positioned it as a foundational tool in NLP.^10^

### Large language models

The transformer architecture underpins the development of LLMs, which promise substantial performance improvements. LLMs have evolved from simpler statistical models into complex neural networks, analysing word sequences to predict subsequent words within a given context. Early LLMs, such as OpenAI’s GPT series, range from GPT (117 million parameters) to GPT-4 (estimated at hundreds of billions of parameters), illustrating the rapid increase in model scale and complexity. Meta's LLaMA models,^31–33^ which come in versions 1, 2, and 3, include parameter sizes ranging from 1 billion to 405 billion, with intermediary models at 3, 8, 13, 70, and 90 billion parameters. While 13 billion and 90 billion models are tailored for multimodal applications, the rest focus on textual tasks, highlighting their versatility and scalability. Additionally, other notable models, such as Mistral,^34^ Qwen,^35^ and others, further enrich the landscape of LLM development, showcasing diverse architectures and application potentials.

LLMs fall into 3 key architectures^36^:

- Encoder-only models (eg, BERT) process entire texts at once, excelling in context-based tasks.^37^
- Decoder-only models (eg, GPT) predict the next word based on preceding words, ideal for text generation.
- Encoder-decoder models (eg, T5^38^ and BART^39^) combine both approaches, balancing context understanding and sequence generation.

Despite their capabilities, LLMs require task-specific fine-tuning to optimize performance.^40^ This process involves exposing the model to relevant data and adjusting its parameters. LLMs, especially GPT-3 and beyond, exhibit potential for “zero-shot” or “few-shot” learning, handling tasks without explicit training, thanks to extensive pre-training on vast datasets. This broad applicability allows models like ChatGPT to generate responses across numerous tasks, though specific fine-tuning may still yield superior results.

The rise of LLMs has spurred novel applications, including advanced language generation and chatbots, redefining human-machine interaction. Their accessibility is enhanced by platforms like Hugging Face’s transformers library,^41^ Google’s TensorFlow,^42^ and Meta's PyTorch.^43^

In medicine, LLMs like Google’s Med-PaLM^44^ have made significant strides, surpassing the pass mark on USMLE-style questions. Med-PaLM 2^45^ further improved accuracy in medical exams and health queries. The MED-PALM M model,^22^ integrating text and imaging data, advances applications like radiomics for tumour characterization and therapy response prediction.

Deploying LLMs demands high-performance hardware (eg, GPUs, TPUs, or specialized accelerators), extensive memory, storage, and high-bandwidth networking to handle massive datasets. These requirements incur significant operational costs and environmental impacts. Organizations are increasingly exploring smaller model architectures and more efficient hardware to reduce these burdens while ensuring sustainable AI development.

Robust version control is also essential in clinical workflows to ensure transparency, reproducibility, and regulatory compliance. Using model-agnostic approaches allows for the seamless integration of updated models, while rigorous sandbox testing, regular audits, and clear documentation help maintain system stability and clinician trust.

## Radiation oncology research applications

### Recent LLM applications in radiation oncology

NLP has been explored for cancer applications,^19^ promising improved care through big data from EHRs and oncology information systems.^46^ Reviews by Yim et al^17^ and Bitterman et al^18^ discuss various NLP applications in radiation oncology, including information extraction from clinical notes,^47^ standardizing treatment planning structures,^48^ and identifying treatment locations.^49^ NLP also aids in toxicity data extraction from clinical notes; however, it suffers in performance with negated symptoms.^50^ Advanced NLP methods have also demonstrated enhancement of cancer registries by directly extracting relevant clinical information from clinical text.^51^ Notably, Khanmohammadi et al^52^^,^^53^ introduced a student-teacher LLM architecture for localized toxicity extraction and iterative prompt refinement, significantly improving accuracy in extracting symptoms and treatments from clinical notes. Their approach leverages automatic prompt optimization through iterative refinement, achieving notable gains in precision and recall while maintaining data privacy and enhancing clinical NLP applications. These applications are summarized in Table 2 and sections “Summarization and retrieval of electronic health records,” “De-identification of sensitive medical data,” “Clinical text mining and decision support,” and “Education and knowledge expansion.”

**Table 2.** Summary of NLP and LLM research applications in radiation oncology.

Application	Task	Details	References
Information extraction	Extracting data from clinical notes	Enhances insights into disease staging, treatment options, and patient outcomes	^17^ ^,^ ^18^ ^,^ ^47^ ^,^ ^52^ ^,^ ^53^
Standardizing treatment planning	Structuring treatment plans	Focused on improving consistency and automation in planning structures	^48^
Identifying treatment locations	Locating treatment areas	NLP identifies anatomical sites for precise treatment targeting	^49^
Toxicity data extraction	Analysing clinical notes for toxicities	Challenges include performance issues with negated symptoms	^50^
Enhancing cancer registries	Extracting registry data	Directly extracts clinical information from unstructured text	^51^
EHR summarization and retrieval	Summarizing and retrieving data	SPeC pipeline improves summary reliability; adapted LLMs outperform experts in reducing documentation burden	^54^ ^,^ ^55^
De-identification of sensitive data	Removing private information from text	DeID-GPT masks private information while preserving text meaning and structure	^56^
Clinical text mining and decision support	Mining text and supporting decision-making	ChatGPT used for named entity recognition and synthetic data generation; multimodal AI enhances decision-making	^57^ ^,^ ^58^
Education and knowledge expansion	Expanding knowledge and training	ChatGPT excels in radiation oncology exams; RadOnc-GPT and OncoGPT enhance diagnostic descriptions and advice	^59–62^

#### Summarization and retrieval of electronic health records

LLMs can enhance electronic health record (EHR) summarization in radiation oncology. The Soft Prompt-Based Calibration (SPeC) pipeline by Chuang et al^54^ addresses output variance, providing reliable summaries. Van Veen et al^55^ found that adapted LLMs outperform medical experts in clinical text summarization, reducing clinicians’ documentation burden.

#### De-identification of sensitive medical data

LLMs, like ChatGPT and GPT-4, offer advanced named entity recognition (NER) capabilities for de-identifying sensitive medical data. Liu et al^56^ proposed DeID-GPT, a framework effectively masking private information while preserving text meaning and structure.

#### Clinical text mining and decision support

LLMs provide insights through clinical text mining. Tang et al^57^ used ChatGPT for NER and relation extraction, generating high-quality synthetic data to fine-tune local models. Ferber et al^58^ demonstrated that multimodal AI systems enhance decision-making by deploying specialized medical AI tools, supporting LLMs as clinical assistants.

#### Education and knowledge expansion

LLMs, like ChatGPT, show promise in medical education. Gilson et al^59^ evaluated ChatGPT’s performance on radiation oncology exams, highlighting its potential for expanding knowledge. RadOnc-GPT,^60^ fine-tuned on Mayo Clinic data, excelled in generating treatment regimens and diagnostic descriptions. Yalamanchili et al^61^ found LLM responses to care questions were on par or superior to expert answers, though readability needed improvement. Jia et al^62^ developed OncoGPT, enhancing accuracy in oncology advice through domain-specific fine-tuning.

### Current shortcomings of NLP models

The rise of LLMs has enabled the development of conversational AI with widespread applications. However, these models often generate hallucinations—irrelevant or incorrect outputs—which is a significant concern in healthcare, where such errors can jeopardize patient safety. Ensuring the reliability and accuracy of LLMs in medical contexts is crucial.

LLMs also exhibit embedded biases,^63^ necessitating collaboration between health professionals and data scientists to prevent encoding historical health disparities. Straw and Callison-Burch^64^ evaluated biases in NLP models used in psychiatry, highlighting significant biases in religion, race, gender, nationality, and sexuality within GloVe^65^ and Word2Vec^66^ embeddings. They emphasized cross-disciplinary collaboration to mitigate health inequalities.

Another issue is model toxicity—producing offensive or discriminatory content.^67^ Evaluations using toxic benchmark datasets are vital to prevent biased outputs and misinformation.^68^ Legal concerns around AI liability in decision-making also necessitate creating reliable, safe, and legally compliant models.^69^ These concerns underscore the need for a robust framework of accountability and transparency in deploying AI tools, ensuring proper review by healthcare providers.

While LLMs have become more accessible and easier to deploy, their comprehensive outputs pose challenges in performance evaluation compared to previous NLP algorithms.^70^ Training and evaluating LLMs require significant computational resources, limiting their use in low-resource environments. Evaluating LLMs’ quality is challenging due to the nuanced nature of natural languages, particularly in clinical settings.

Rigorous evaluation and validation are essential to ensure that NLP models are reliable and safe for clinical use. The rapid development and deployment of these technologies necessitate a comprehensive evaluation before clinical implementation. The next section proposes a checklist for step-wise evaluation of NLP models before their deployment in radiation oncology.

## Framework for clinical implementation

**Figure 2.**
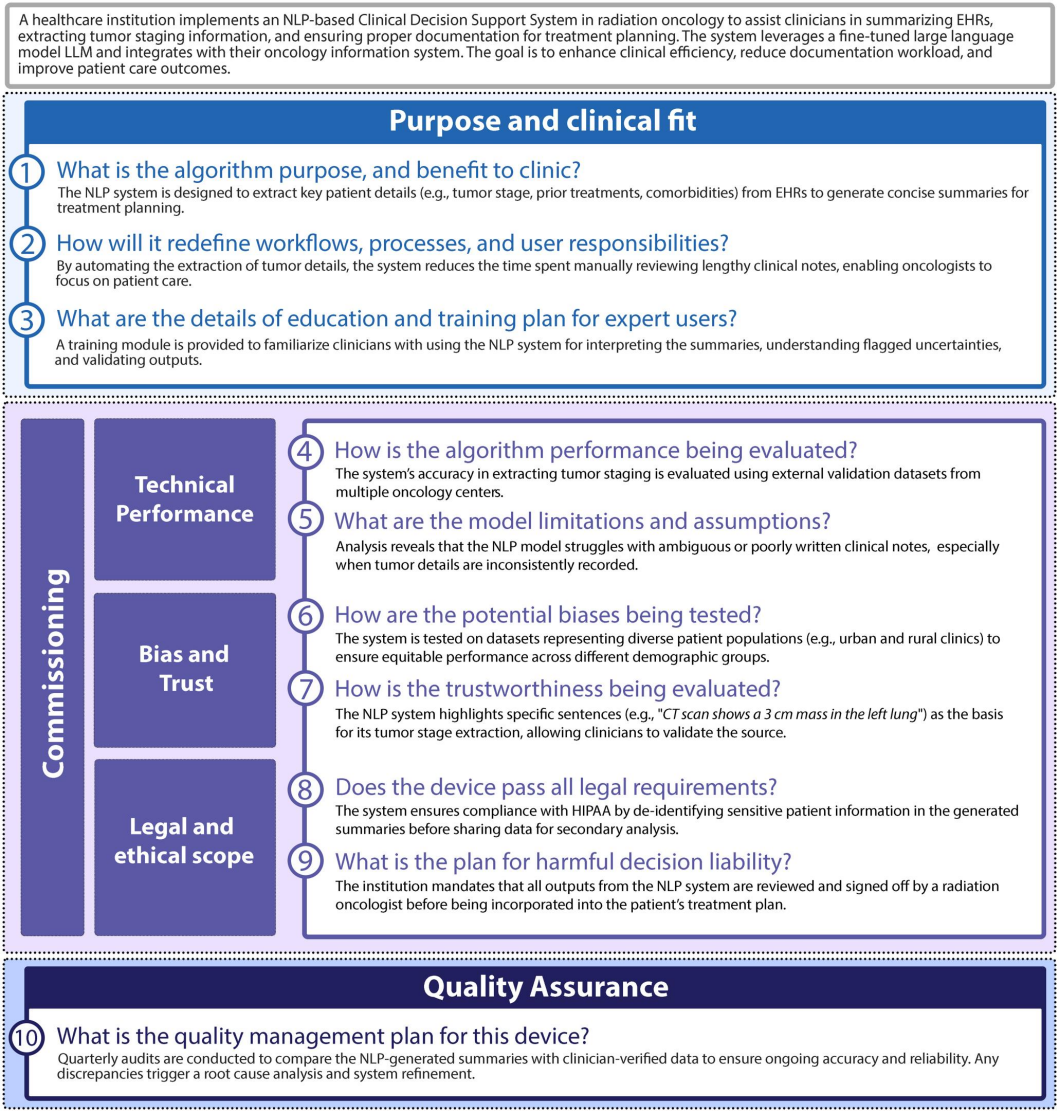
Framework for assessing natural language processing algorithms before they are deployed in clinical settings. The framework consists of several categories and questions that can help evaluate the objectives, outcomes, limitations, reliability, validity, and quality control measures of the algorithms, as well as their implications for clinical practice and patient safety.

This section presents a comprehensive framework for the clinical implementation of NLP systems in radiation oncology. The framework consists of 3 main components: (1) evaluation of the purpose and clinical fit of the NLP system, (2) commissioning of the NLP system, and (3) quality assurance of the NLP system. The commissioning section includes sub-sections on technical performance, bias and trust, and legal and ethical scope. A graphical overview of the framework and a checklist of relevant questions for the clinical commissioning team are provided in Figure 2.

### Purpose and clinical integration

Clinical implementation of an NLP model begins with clearly defining its purpose by outlining the clinical problem, context, chosen solution, and anticipated benefits. In radiation oncology, innovations are first evaluated for their potential to improve efficiency and efficacy. For example, an NLP model might extract tumour staging data from EHRs to support treatment planning or generate concise patient record summaries for tumour board discussions. Although the problem and solution can be broadly defined, they must include concrete, testable components to ensure reliability before routine use.

Beyond purpose, it is essential to articulate the expected impact. An NLP tool that automates toxicity data extraction, for instance, should be compared against manual reviews to verify accuracy. Similarly, automating patient record summarization can reduce clinicians’ review time, streamline workflows, and allow more focus on direct patient care. Effective planning should also address how the technology will augment clinical expertise through comprehensive education and training, ensuring that users can validate and fully leverage the system.

### Commissioning

#### Technical performance

NLP algorithm technical performance is evaluated on specific development datasets that are reflective of the expected clinical performance. The development dataset is usually divided into 3 subsets: train, development/validation, and test. The train set is used to train the initial algorithm, the validation set is used to adjust the algorithm parameters for specific tasks, and the test set is used to measure the algorithm performance. An important consideration for the implementation team is to know that the testing subset should be independent of the training and tuning subsets. The level of this independence can be categorized as external validation, internal validation, and cross-validation. External validation, where the testing subset comes from a different source than the training and tuning subsets, is the most rigorous evaluation method. Internal validation, where the testing subset is separated from the training and tuning subsets within a single source dataset, is the next best method. Cross-validation, where the testing subset overlaps with the training and tuning subsets, is a weaker method due to potential bias.^71^ Nested cross-validation, however, serves as a more conservative alternative to traditional cross-validation, thereby reducing the risk of information leakage among different sample sub-cohorts.^72^ In this method, cross-validation is performed within the training set to choose the model parameters, and an external cross-validation loop is used to estimate the error of the chosen model.

Once the dataset selection is validated, the algorithm performance evaluation can be appraised. Generally, performance evaluation is closely associated with the NLP task at hand. NLP tasks can be broadly categorized into classification tasks, NER, entity abstraction, summarization tasks, and question-answering tasks.^73^ For classification tasks, as well as for NER and entity abstraction, metrics such as positive predictive value, negative predictive value, area under the receiver operating characteristic curve, and F-measures (harmonic mean of precision and recall) are used to assess sensitivity and specificity, with likelihood ratios (positive and negative) providing additional clinical context. For summarization tasks, the Rouge^74^ metrics measure the overlap between machine-generated and human-generated summaries, while question-answering tasks are evaluated using the Bilingual Evaluation Understudy (BLEU)^75^ metric, assessing the quality of translated text by comparing it to a set of high-quality reference translations. Choosing the appropriate performance metric is essential for benchmarking and ensuring clinical relevance.

#### Bias and trust

**Figure 3.**
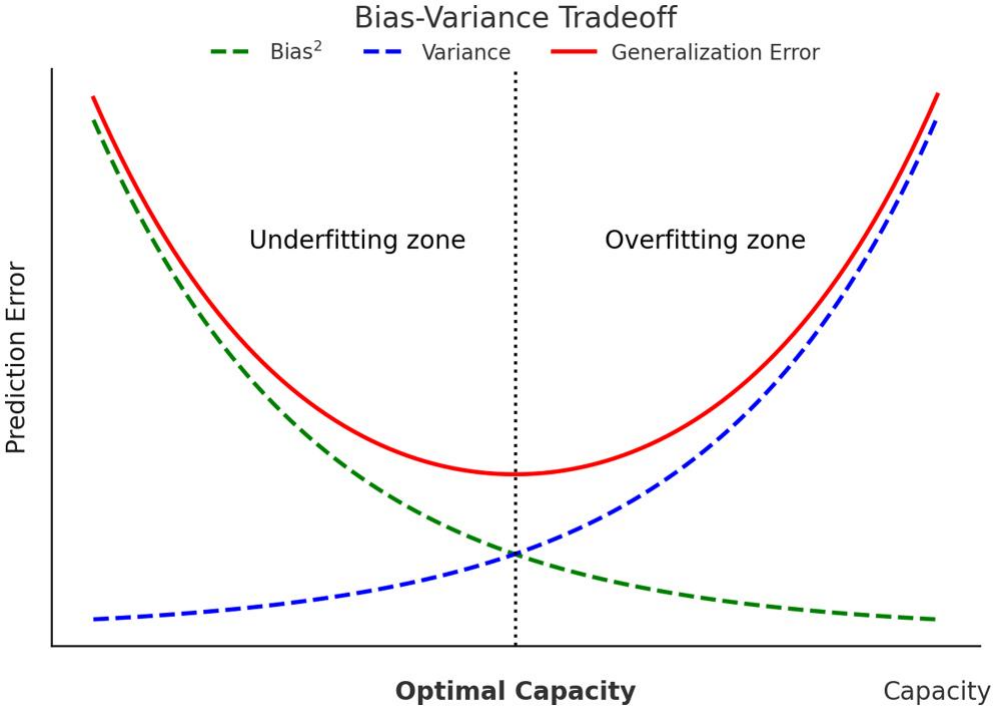
This figure illustrates the bias-variance tradeoff as a function of model capacity. The *x*-axis represents model capacity, ranging from low to high, while the *y*-axis represents Prediction Error, which quantifies the expected discrepancy between model predictions and true values (eg mean squared error [MSE]). Three key trends are displayed: bias^2^ (descending curve), variance (ascending curve), and generalization error (U-shaped curve). In the area of low capacity, the model is prone to underfitting, represented by high bias and low variance. As we move towards higher capacities, the model starts to overfit, demonstrated by low bias but high variance. The optimal model capacity is depicted where the generalization error is minimized, balancing bias and variance. This point represents the most effective model complexity for preventing both underfitting and overfitting, thus achieving optimal performance on unseen data.

Technically, bias can be minimized by optimizing for the lowest generalization error, which is measured by the out-of-sample error and the gap between ground truth and prediction.^76^ This gap can be caused by model inaccuracy, sampling error, or noise. The generalization error can be reduced by choosing the right algorithm, tuning it well, and using large cohorts of diverse data. However, tuning of the model should be balanced as overfitting to variance also results in poor generalization error and poor outputs.^77^ Therefore, the optimal model should capture the data’s meaningful patterns without being overfitted, as demonstrated in Figure 3. Beyond the technical principle of minimizing generalization error, the algorithm should have a low bias in the domains of statistical bias, algorithmic bias, and societal bias.^78^

Statistical bias arises when training data does not represent the broader population—for example, a cancer outcome model trained mainly on urban hospital data may underperform for rural patients. This can be mitigated by using diverse datasets with sufficient variance and samples to capture rare conditions. Algorithmic bias stems from design flaws—such as word embeddings linking “doctor” mostly with male pronouns and “nurse” with female pronouns—reflecting historical oversights in considering biological, environmental, and social factors.^79^ Minimizing this bias requires carefully designed training objectives, fairness-aware algorithms, and debiasing techniques.^80^ Lastly, societal bias reflects deep-rooted biases in the data that can lead to unequal outcomes by associating conditions disproportionately with certain demographic groups.^81^ Addressing all these domains is critical to ensure that NLP models perform fairly across diverse patient populations.^78^

To address these biases in practice, several tools and frameworks have been developed for detecting and mitigating bias in NLP models. TCAV (Testing with Concept Activation Vectors)^82^ is a Google-developed framework that helps to interpret and analyse the behaviour of machine learning models, while audit-AI (https://github.com/pymetrics/audit-ai) and IBM's AI Fairness 360 (AIF360)^83^ examine machine learning models to detect race, gender, location, and other sources of bias and discrimination. While these tools may be helpful overall, the testability for narrow use cases may be restrictive. If the algorithm under consideration has resulted in manuscripts and clinical trials, the CLAIM (Checklist for Artificial Intelligence in Medical Imaging),^84^ Consort-AI, and CLAMP (Clinical Language Annotation, Modeling, and Processing)^85^ publications can be utilized and translated to ensure adequate study organization, scientific reporting, and robust performance testing including sub-group representation.^80^ Lastly, it is important to remember that the original data used for the development and testing of NLP pipelines may not be representative of an institution’s local data. Therefore, the clinic must ensure that the algorithm ranks high on the fairness scale when applied to diverse patient populations, and as such, the tools and concepts mentioned above can aid in clinical implementation.

Clinician trust in the NLP algorithm should be evaluated next on the path towards clinical implementation. Trust is a psychological mechanism that deals with the uncertainty between known and unknown, where algorithm transparency, predictability, and fairness can play a large role in the trustworthiness of clinicians.^86^ Algorithm fairness has been discussed in the previous section, where minimization of bias is critical. The transparency of the algorithm is related to the explainability and interpretability of the results, so that the algorithm can map the output to a selection of inputs.^87^ In general, the more advanced an algorithm is, the lower the explainability.^88^ However, to promote trust, explainability and interpretability are increasingly being incorporated in the more advanced algorithms.^89^ Beyond the explainability, the trust in the algorithm is largely based on the predictability of outcomes, especially when faced with conflicting inputs.

Similar to the interpretability approaches for machine learning described, NLP methods also fall into the categories of model-specific and model-agnostic interpretability.^90^ For instance, the attention mechanism itself provides some level of model-specific interpretability. They offer a glimpse into the workings of the model by illustrating the importance of different words or phrases in the input for the model’s decision-making.^8^ This way, clinicians can potentially understand which parts of a patient's history or report the algorithm deemed significant. On the model-agnostic side, techniques such as LIME (https://c3.ai/glossary/data-science/lime-local-interpretable-model-agnostic-explanations/) (Locally Interpretable Model-agnostic Explanations) and SHAP (SHapley additive exPlanations)^91^ have also been applied to NLP. For example, LIME can provide insight into the model's decisions by perturbing the input and observing the model's output, thereby explaining individual predictions.^92^ SHAP values can provide a global view of feature importance across all predictions, demonstrating how much each feature (or words in a textual modality) contributes to the model's decisions. For example, a study by Khanmohammadi et al^93^ utilized SHAP for interpretability in clinical NLP tasks, demonstrating the most significant sound features in predicting foetal biological sex using Phonocardiogram signals. These methods enhance the transparency of NLP algorithms and provide clinicians with a better understanding of how the algorithms arrive at their conclusions. Published NLP algorithms are designed to work on specific tasks, with the embedded assumption that the training and test data are generated from a similar statistical distribution. This assumption may easily be violated in clinical scenarios, where input data may not match the statistical assumptions, and the algorithm's stability under these inputs will be directly associated with the clinician's trust in them. To test for the predictability of outcomes, and an algorithm's stability, the concept of adversarial testing can be used to estimate algorithm performance under unstable inputs.^94^ There are several methods for performing adversarial testing, with the evasion method perhaps being the most suitable for testing the edges of the NLP model.^95^ This testing would encompass actively modifying input data that represents the most extreme clinical scenario and analysing the algorithm output for (1) transparency—does the model explain or interpret the outputs to specific inputs? (2) predictability—does it show the same result every time? (3) fairness—does the output represent a sensible answer that is rooted in the representation of all sub-groups within the clinical setting?^96^

#### Legal and ethical considerations

Legal and regulatory frameworks ensure the safe and ethical use of AI algorithms in healthcare. These address the potential risks and challenges and cover 3 main aspects of AI development and deployment, namely, how medical devices are regulated, how health data privacy is protected, and how liability is assigned for any harm caused by faulty, erroneous, or unsafe algorithm recommendations.^97^ Algorithm regulation strictly follows the Food and Drug Administration (FDA) guidelines in the United States, and significant progress has been made by the regulatory agency in defining standards and guiding principles for AI algorithms. NLP algorithms, being a subclass of AI, will fall under the category of Software as a Medical Device (SaMD). The FDA has established a regulatory framework IN 2019 for these devices (https://www.fda.gov/files/medical%20devices/published/US-FDA-Artificial-Intelligence-and-Machine-Learning-Discussion-Paper.pdf), which proposes a risk-based regulation based on the intended use of the device and the patient’s risk from inaccurate output. In 2021, the FDA proposed an action plan and guiding principles in collaboration with Canada and UK to ensure safe, effective and quality SaMD use (https://www.fda.gov/medical-devices/software-medical-device-samd/artificial-intelligence-and-machine-learning-software-medical-device, https://www.fda.gov/medical-devices/software-medical-device-samd/good-machine-learning-practice-medical-device-development-guiding-principles). Recent publications have discussed the regulation of LLM-based chatbots as medical devices.^98^ Therefore, it is critical to understand the level of regulation for the device under consideration and the regulatory implications of the FDA to ensure effective clinical use.

Health data privacy is critical as LLMs become more integrated into clinical workflows. While HIPAA provides a foundational framework for protecting patient health information, it has limitations with modern AI technologies. As noted by Rezaeikhonakdar et al,^99^ many AI tools and chatbots are not classified as HIPAA-covered entities or business associates, meaning patient interactions with systems like ChatGPT or Google Gemini may fall outside HIPAA’s protections. Even when these systems claim HIPAA compliance, risks such as re-identification of de-identified data remain, as highlighted in cases like Dinerstein v. Google,^100^ and these risks are compounded when large tech companies cross-reference extensive data resources, potentially compromising patient confidentiality.^101^^,^^102^ Moreover, Marks and Haupt^103^ emphasize that HIPAA compliance alone may not protect patient data from misuse; chatbots can prompt users to disclose sensitive information, which may then be leveraged for unauthorized data sharing, targeted advertising, or discriminatory practices in insurance and employment. To mitigate these risks, developers must adopt rigorous data governance and transparent practices in data usage. Clinicians also play a key role by limiting the input of sensitive data into these tools and ensuring they act as responsible data stewards, understanding how these systems manage and process information. Informed consent is essential, with patients needing to know how their data will be used, stored, and protected, thereby building trust and upholding ethical principles of autonomy and respect for patients.^104^ Additionally, robust liability systems must be established to address any harm caused by NLP algorithms, ensuring accountability and patient safety.^102^^,^^105^

#### Quality assurance

The technical and clinical performance of the model is estimated during the commissioning process. A Quality Management Program (QMP) details the performance tests, frequency of testing, expected outputs, and plan of action for inadequate performance. Further, it should include a quality improvement section, where the inadequate performance of the algorithm under routine use can be evaluated and discussed in detail to ensure safe and quality clinical care. As such, QMP can be divided into routine quality assurance and case-specific testing for quality improvement.^106^ Routine quality assurance must be performed periodically with a stable reference dataset every time to ensure the stability of outputs. Ideally, the reference data is a subset of the data that is utilized during the commissioning process and is representative of routine clinical data and use cases. This step should also be used for the clinical release of a device after downtime or minor changes, such as changes in computation hardware. A comprehensive data logging system is recommended for the structured collection of algorithm input, output, and stability. This should be a pivotal piece of the quality improvement component, whereby the unexpected performance by the algorithm can be traced back to the inputs, and root cause analysis can be performed to uphold safe and quality-driven clinical care. A review of case-specific performance further allows for the identification of model limitations that can facilitate future model revisions.^106^ We believe that new and emergent use cases for the algorithm should be evaluated fully by testing purpose, clinical fit, commissioning, and quality assurance plan as outlined in section “Framework for clinical implementation” This rigorous step will ensure that the algorithm performance is suitable to ensure safe and high-quality clinical care.

#### Real-world validation of the framework for clinical implementation

The proposed framework is validated by research studies employing similar methodologies. Walker et al^49^ developed an NLP tool to standardize free-text treatment site documentation in radiation oncology EMRs, using domain-specific dictionaries and error correction to achieve superior precision and recall, thereby aligning with the framework’s emphasis on defining purpose and clinical fit. Similarly, Hong et al^50^ demonstrated the commissioning process with an NLP pipeline for automating toxicity data abstraction, achieving high accuracy for toxicities like radiation dermatitis and fatigue while addressing challenges with negated symptoms to guide iterative improvements in performance metrics, transparency, and fairness. Furthermore, Mathew et al^107^ integrated NLP and machine learning into an incident learning system, streamlining workflows, promoting a safety culture, and supporting scalability through open-source pipelines and standard taxonomies, which reinforces the framework’s focus on routine and case-specific quality assurance. Together, these studies confirm that a structured approach encompassing purpose, clinical integration, commissioning, and quality assurance can successfully deploy NLP systems in radiation oncology, enhancing both operational efficiency and clinical outcomes while addressing key implementation challenges.

## Conclusion

In this article, we have discussed the recent advances and applications of NLP in radiation oncology. NLP is a powerful tool that can transform unstructured clinical narratives into structured data for medical AI systems. NLP models utilizing LLMs and based on self-attention transformer architecture can perform multiple domain-specific tasks through transfer learning, which reduces the need for large annotated data sets and training burdens. These models demonstrate good performance. However, before these models can be implemented and used in routine clinical care, they need to be rigorously evaluated for their validity, functionality, viability, safety, and ethical use. We have also proposed a framework that radiation oncology clinicians can use to assess the suitability of NLP models for their needs. The checklist aptly discusses key areas of algorithm training, tuning, transparency and interpretability, bias, and fairness, as well as legal and ethical concerns. Overall, these novel NLP techniques can enable the creation of more advanced AI models, which can improve patient outcomes and expedite the progress of precision medicine in radiation oncology under appropriate ethical and technical constraints.
